# Fluorescence Lifetime Imaging Microscopy (FLIM) reveals spatial-metabolic changes in 3D breast cancer spheroids

**DOI:** 10.1038/s41598-023-30403-7

**Published:** 2023-03-03

**Authors:** Kavon Karrobi, Anup Tank, Mohammad Ahsan Fuzail, Madhumathi Kalidoss, Karissa Tilbury, Muhammad Zaman, Jacopo Ferruzzi, Darren Roblyer

**Affiliations:** 1 Department of Biomedical Engineering, Boston University, Boston, MA 02215 USA; 2 Department of Chemical and Biomedical Engineering, University of Maine, Orono, ME 04469 USA; 3 Department of Biomedical Engineering, The University of Texas at Dallas, Richardson, TX 75080 USA

**Keywords:** Breast cancer, Cancer metabolism, Extracellular matrix, Cell invasion, Cellular imaging

## Abstract

Cancer cells are mechanically sensitive to physical properties of the microenvironment, which can affect downstream signaling to promote malignancy, in part through the modulation of metabolic pathways. Fluorescence Lifetime Imaging Microscopy (FLIM) can be used to measure the fluorescence lifetime of endogenous fluorophores, such as the metabolic co-factors NAD(P)H and FAD, in live samples. We used multiphoton FLIM to investigate the changes in cellular metabolism of 3D breast spheroids derived from MCF-10A and MD-MB-231 cell lines embedded in collagen with varying densities (1 vs. 4 mg/ml) over time (Day 0 vs. Day 3). MCF-10A spheroids demonstrated spatial gradients, with the cells closest to the spheroid edge exhibiting FLIM changes consistent with a shift towards oxidative phosphorylation (OXPHOS) while the spheroid core had changes consistent with a shift towards glycolysis. The MDA-MB-231 spheroids had a large shift consistent with increased OXPHOS with a more pronounced change at the higher collagen concentration. The MDA-MB-231 spheroids invaded into the collagen gel over time and cells that traveled the farthest had the largest changes consistent with a shift towards OXPHOS. Overall, these results suggest that the cells in contact with the extracellular matrix (ECM) and those that migrated the farthest had changes consistent with a metabolic shift towards OXPHOS. More generally, these results demonstrate the ability of multiphoton FLIM to characterize how spheroids metabolism and spatial metabolic gradients are modified by physical properties of the 3D ECM.

## Introduction

An abnormal extracellular matrix (ECM) is a key component of the breast tumor microenvironment and plays a critical role with cancer development, progression, and metastasis^1^. Among various ECM constituents, collagen content has been associated with mammographic density, an established risk factor for breast cancer^2^. Tumor cells can mechanically sense the physical properties of its environment and engage with the ECM through integrins and affect downstream signaling to promote a more invasive phenotype^3^. For example, the mechanosensing YAP/TAZ pathway, modulates several downstream signaling cascades in stiff tumor microenvironments to increase cellular invasion and migration in addition to modulating metabolic pathways^4,5^.

Cancer cells reprogram their energy metabolism to support their uncontrolled growth and proliferation^6^. An extremely conserved modification across many cancer types is the Warburg effect, also called aerobic glycolysis^7,8^, which is the tendency for cancer cells to shift towards glycolysis and fermentation over oxidative phosphorylation and respiration even in the presence of oxygen. The shift serves to sustain extensive cell growth and replication. While the Warburg effect has been extensively characterized, it is only one of the many metabolic perturbations observed in tumors.

Recent evidence suggests that proliferating cancer cells have different metabolic profiles than migrating cancer cells^9^. Cancer cell migration is a pivotal step of the metastatic cascade. Understanding the phenotypic mechanisms that drive tumor cells to migrate and eventually metastasize is critical for improving patient outcomes^6^. The metabolic plasticity of cancer cells sustains their ability to dynamically adjust from a proliferative to migratory phenotype^9^. Migratory cells require a significant amount of energy to support cytoskeletal modifications and ECM remodeling. Importantly, the metabolic demand for remodeling the ECM can depend on the physical properties of the ECM^10^. Cancer cells can follow leader–follower invasion dynamics where leading migratory cells utilize more ATP, as they engage and modify the ECM, than the follower migratory cells^11^. The metabolic differences in leader versus follower cells and their importance in invasion highlights the need for spatially resolved methods to measure metabolic profiles to develop effective therapies targeting migrating cancer cells^12^.

Fluorescence Lifetime Imaging Microscopy (FLIM) is a non-invasive, spatially resolved label-free imaging modality that measures the fluorescence decay of fluorophores and can be used to measure the metabolic profile of live cancer cells^13–15^. Multiphoton FLIM enhances both axial resolution and depth of penetration, thus enabling sub-micron sensitivity throughout 3D tumor spheroid models that are hundreds of microns in diameter and are embedded within ECM analogs that are millimeters in thickness. Consequently, imaging fluorescence decays in invading tumor spheroids would not be possible using single photon techniques such as confocal microscopy^16^. The metabolic coenzymes nicotinamide adenine dinucleotide (NAD(P)H) and flavin adenine dinucleotide (FAD) are autofluorescent and their respective fluorescence decay rates depend on if the coenzymes are free versus bound^14^. The relative proportions of free and bound NAD(P)H or FAD can be used to infer whether glycolysis or oxidative phosphorylation are the metabolic pathways used by cancer cells to fuel various cellular processes and have been shown to correlate with conventional metabolic assays such as mass spectrometry and seahorse flux analysis^15,17–23^. Similarly, the ratio of the bound NAD(P)H to bound FAD, the Fluorescence Lifetime Imaging Redox Ratio (FLIRR), is a promising optical biomarker to determine a cell’s primary metabolic pathway with higher FLIRR values often consistent with a shift towards oxidative phosphorylation and lower values often associated with a shift towards glycolysis^24^. A cancer cell’s metabolic profile can provide critical information about its phenotype such as the differences in leader or follower characteristics^11^, or how the cancer cell is engaging with and potentially modifying the ECM^19^. Other fluorescence intensity-based methods have been developed that are sensitive to other metabolic parameters such as the ATP/ADP ratio^11^ or the NADH/NAD+ ratio^25^, but require genetically encoded receptors. In contrast, FLIM can provide a label-free method to image and quantify a cells’ metabolic profile and investigate the spatial relationship of cells with different metabolic demands in the context of cellular microenvironment.

Prior studies investigating the effect of the ECM on cancer cells’ metabolism have had two main limitations. First, previous studies using FLIM to examine cancer cell metabolism have mainly been done in 2D cultures^19^, which are unable to accurately recapitulate the 3D structural mechanical features of the ECM^26^. Additionally, epithelial cancer cells tend to be more glycolytic in 2D culture than in a 3D environment and display less metabolic plasticity^27^. Secondly, other studies have used metabolic assays that provide molecular specificity but require bulk samples preventing the examination of the spatial metabolic trends particularly important in the case of cancer cell migration^28^. The work conducted by Liu et al., for example, determined that stiffness of the ECM modulated the migratory and proliferative properties of 3D breast cancer cultures. They performed RNA-seq which resulted in bulk spheroid genetic enrichment scores for metabolic pathways. Importantly, the authors demonstrated that stiffer environments induced a migratory phenotype that correlated with an overall enrichment in oxidative phosphorylation. However, bulk RNA-seq is unable to characterize the spatial metabolic profiles associated with the complex metabolic phenotypes within 3D spheroids. The characterization of single-cell metabolic phenotypes in migrating cells is therefore needed to further explore the hypotheses at the intersection of cellular metabolism and spatial dynamics.

Here we present a study in which we used FLIM to image the spatial metabolic profile during migration of breast cancer spheroids embedded in 3D collagen gels at two different collagen concentrations (1 mg/ml and 4 mg/ml) across two different time points (Day 0 and Day 3). We imaged two different human-derived breast cell lines: MCF-10A, a mammary epithelial cell line^29^, and MDA-MD-231, a highly invasive breast cancer cell line^30^. Collagen concentrations of 1 and 4 mg/ml were chosen to mimic, respectively, low-density ECM and high-density ECM, which have been recently shown to induce radically different migratory phenotypes in these cell lines over the course of 3 days of culture^31^. For this reason, we investigated the differences in migration and spheroid area for each cell line from Day 0 to Day 3, and the changes in the overall metabolic profile of the two breast cancer cell lines. Finally, the metabolic spatial maps of each spheroid were analyzed to probe any spatial metabolic relationships as a function of distance from the ECM or migration distance.

## Methods

### Cell lines and culture media

Metastatic MDA-MB-231 and non-tumorigenic MCF-10A human breast cell lines were purchased from American Type Cell Culture Collection (ATCC) and cultured using standardized media and conditions as previously described^31^. Briefly, MDA-MB-231 cells were cultured in DMEM (Corning, No. 10013CV), supplemented with 10% fetal bovine serum (ATCC, No. 302020). MCF-10A cells were cultured in DMEM/F-12 (ThermoFisher, No. 11330032) supplemented with 5% horse serum (Invitrogen, No. 16050122), 20 ng/ml EGF (Peprotech, AF10015; ThermoFisher, No. 10605HNAE), 0.5 mg/ml hydrocortisone (Sigma-Aldrich, No. H0888), 100 ng/ml choleratoxin (Sigma-Aldrich, C8052), 10 mg/ml insulin (Sigma-Aldrich, No. I1882). Both media recipes contained 1% penicillin/streptomycin (ATCC, No. 302300; ThermoFisher, No. 15140122). Cells were maintained at 37 °C and 5% CO_2_ in a cell culture incubator.

### Preparation of polydimethylsiloxane (PDMS) wells

Cylindrical PDMS wells (9 mm in diameter) were created inside 35 mm glass-bottom petri dishes (MatTek, No. P35G-0-10-C) using 3D printed self-centering cylinders as previously described^32^. Briefly, 1 ml of a liquid PDMS mixture (Dow Corning, Midland, MI) with a 10:1 weight ratio of silicon elastomer to curing agent was deposited inside the dishes and around the cylinders and cured at 50 °C for 2 h. After removal of the cylinder, the resulting cylindrical well was cleaned from PDMS residues and coated using poly-d-lysine (Sigma-Aldrich, St. Louis, MO) and glutaraldehyde, which provided an anchoring layer for collagen to avoid gel floating. After rinsing with 1 × PBS, the PDMS wells were sterilized under ultraviolet (UV) light for 30 min.

### Spheroid formation

MDA-MB-231 and MCF-10A spheroids were generated as previously described^31^. Briefly, spheroids were generated by seeding approximately 1 × 10^3^ cells in each of the 96 wells of an ultra-low attachment plate (Corning, No. 7007) and allowed to form for 48 h in the presence of 2.5% v/v Matrigel. Once formed, individual spheroids surrounded by 5 µl of media were transferred onto coverslips inside PDMS wells (9 mm in diameter) created in 35 mm glass-bottom petri dishes (one spheroid per dish). Each spheroid was covered by 195 µl of ice-cold, rat-tail collagen I solution to achieve a total volume of 200 µl and a specific collagen concentration in each well. Collagen solutions were prepared by mixing acid-solubilized collagen I (Corning, No. 354249) with equal volumes of a neutralizing solution (100 mM HEPES buffer in 2 × PBS). The desired collagen concentration was reached by adding adequate volumes of 1 × PBS. Collagen solutions at different concentrations (1 and 4 mg/ml) polymerized for 1 h at 37 °C. The cell culture plates were rotated every minute for the first 10 min of polymerization to guarantee full embedding of the spheroid within the 3D collagen matrix. Finally, 2 ml of culture media (phenol-free, 50:50 v/v of MDA-MB-231 media to MCF-10A media) was added and the 3D organotypic culture was placed inside the incubator until taken out for FLIM measurements.

### Multiphoton fluorescence lifetime imaging microscopy (FLIM)

Label-free multiphoton FLIM was used to monitor intracellular NAD(P)H and FAD fluorescence intensity and lifetimes of MDA-MB-231 and MCF-10A spheroids completely embedded in 3D collagen gels at different densities (1 and 4 mg/ml) over time (days 0 and 3). These spheroids were imaged at a depth of ~ 1–2 mm from the surface of the collagen gel in a single optical section. All spheroid samples were allowed to incubate in fresh phenol-free 50:50 culture media for a minimum of 3 h prior to imaging. FLIM was performed using an upright multiphoton microscope (Bruker, Ultima Investigator) equipped with a stage top incubator (Tokai Hit, STXF-UKX-SET) to maintain samples at 37 °C and 5% CO_2_ with humidity during imaging.

**Figure 1:**
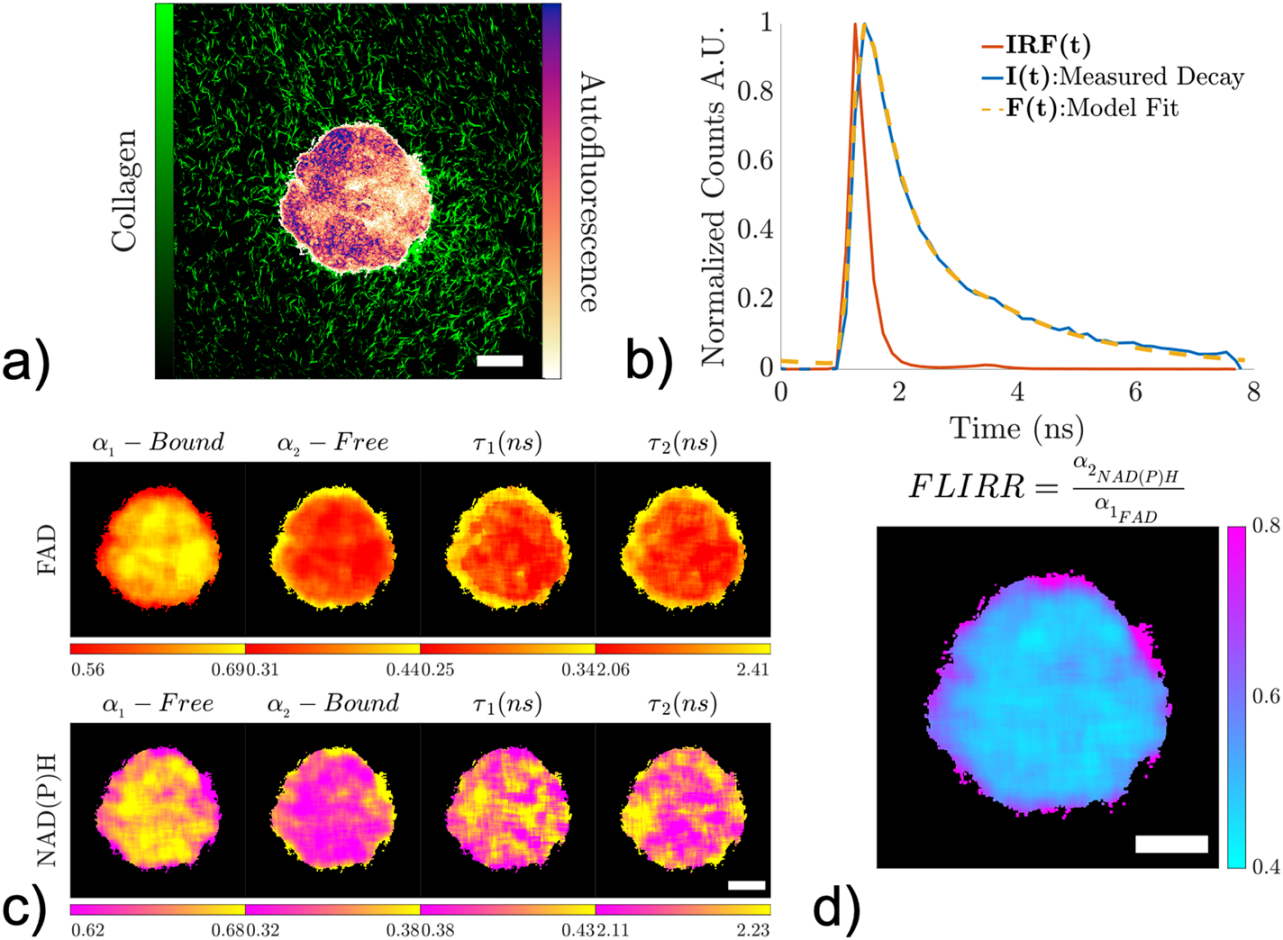
FLIM methodology and processing. (**a**) An overlay of time-integrated NAD(P)H autofluorescence intensity from a MCF-10A breast cell spheroid and second harmonic generated collagen signal from a 4 mg/ml collagen gel imaged at Day 3. (**b**) Representative example of the measured decay signal I(t), which is a convolution of the measured impulse response function IRF(t) and the autofluorescence decay. The model fit F(t) is computed by convolving IRF(t) with a bi-exponential fluorescence decay model and comparing to I(t) using a nonlinear least squares fitting routine. (**c**) Images of the four parameters (${\boldsymbol{\alpha }}_{1}$, ${\boldsymbol{\alpha }}_{2},\boldsymbol{ }{{\varvec{\tau}}}_{1}$*,* ${{\varvec{\tau}}}_{2}$) extracted from the nonlinear least squares fitting procedure for both FAD and NAD(P)H. ${{\varvec{\tau}}}_{1}$ and ${{\varvec{\tau}}}_{2}$ values are shown in units of nanoseconds. (**d**) A representative image of Fluorescence Lifetime Imaging Redox Ratio (FLIRR) values in a MCF-10A spheroid, which is obtained as the ratio of bound NAD(P)H to bound FAD from part (**c**). Scale bars (white) indicate 100 µm.

A femtosecond titanium:sapphire tunable laser (Spectra-Physics, InSight X3) was used as the excitation source with a quarter-waveplate in the excitation path to circularly polarize the incident beam at the sample plane. The laser was tuned to either 760 or 880 nm for two-photon imaging of NAD(P)H or FAD, respectively. For collagen imaging, through second harmonic generation (SHG), the laser was tuned to 1050 nm. The emission was detected in a non-descanned geometry through a 16× long working distance (3 mm) water immersion objective (0.8 NA) (Nikon, CFl75 LWD 16X W), and separated by a dichroic mirror (Chroma, 700 nm long-pass). A 720 nm short-pass filter blocked residual excitation wavelengths in the detection path. NAD(P)H and FAD fluorescence emission was collected using a 440 ± 40 or 550 ± 50 nm bandpass filter (Chroma), respectively. A GaAs photon counting photomultiplier tube (Hamamatsu, H10770PB-50) with a time-correlated single photon counter (Becker & Hickl, SPC-150) was used to collect the temporal decays of NAD(P)H and FAD autofluorescence (120 s collection time per frame, 256 temporal bins per pixel). Images were collected at a scanning resolution of 0.8 µm/pixel and 10 µs pixel dwell time using 1024 × 1024 pixels (Fig. 1a).

SHG imaging of randomly oriented collagen fibers in the spheroid-embedded collagen samples was used to estimate the instrument response function (IRF) for both NAD(P)H and FAD FLIM configurations. To collect the IRF, the laser was tuned to 760 nm with a 375 ± 30 nm bandpass filter for NAD(P)H analysis and tuned to 880 nm with a 440 ± 40 nm bandpass filter for FAD analysis, which had on average full width half maximums of 339 ± 8 ps and 396 ± 16 ps, respectively. SHG images (Fig. 1a) of the collagen environment surrounding the embedded spheroids were acquired with the laser tuned to 1050 nm and a 525 ± 35 nm bandpass filter for collection. There was a total of 8 conditions (2 cell lines × 2 collagen densities × 2 days), with each condition having n = 3 samples. Graphical data was generated by pooling all 3 samples for each condition.

### Image segmentation

Binary masks were generated by processing NAD(P)H intensity images (Fig. 1a), which were derived from the NAD(P)H lifetime data for each sample by summing the NAD(P)H temporally binned photon counts in each pixel. Two different binary masks methods were developed in MATLAB (MathWorks, R2021a) to process NAD(P)H intensity images. One method was developed to process images that contained either full or partial spheroids, and another to process images that only contained migrating cells, as one image processing pipeline was not sufficient to accurately segment both image types. For samples with full or partial spheroids, the intensity images were normalized between 0 and 1 and then a two-class Otsu’s method^33^ was used to binarize and segment spheroids and cells from the background. The binarized and segmented images were then dilated using the built-in MATLAB functions *imdilate* and *strel* with a disk-shaped kernel (3-pixel radius) to fill in the shape of the segmented spheroids and cells. The built-in MATLAB function *imfill*^34^ was then used to fill in any remaining gaps, followed by clearing of extraneous pixels in contact with the image borders using the built-in MATLAB function *imclearborder*.

For samples with only migrating cells, binary masks were generated from intensity images using a modified procedure to segment tumor vasculature^35^. These steps consisted of (1) a white top-hat transform, with the Matlab function *imtophat* to enhance smaller elements of the image, (2) normalizing images between 0 and 1, (3) applying an anisotropic diffusion filter^36–38^ to reduce noise in the images while preserving cell edges, and (4) finally a multiscale Hessian filter^39^ was applied to the images along with another top-hat transform to yield an intensity image with enhanced contrast. The image was binarized using a locally adaptive threshold^40^ with the MATLAB function *adaptthresh*, and refined using active contour segmentation^41^ with the MATLAB function *activecontour* by inputting the contrast enhanced intensity image from step (4) and the binarized image.

### FLIM image analysis

Binary masks were imported in MATLAB and used to isolate and analyze NAD(P)H and FAD lifetime data from spheroids and cells based on a previously described and validated fitting routine^42^. Briefly, each pixel within a given image (NAD(P)H or FAD) contained a time decay of fluorescence intensity $I\left(t\right)$ (Fig. 1b). To maximize the total photon count per pixel in the temporal decays used during the fitting routine, lifetime data in each image was summed over space (20 × 20 pixels moving window) and binned in time (256 temporal bins to 64 temporal bins) after applying the corresponding binary mask. The resulting lifetime images were then used to extract lifetime parameters for NAD(P)H and FAD. A bi-exponential fluorescence decay model was used to describe temporal decays of fluorescence intensity:

$$m\left(t\right)= {\alpha }_{1}{e}^{-\frac{t}{{\tau }_{1}}}+ {\alpha }_{2}{e}^{-\frac{t}{{\tau }_{2}}}$$

where ${\tau }_{1}$ and ${\tau }_{2}$ represent the fast and slow lifetime components (in ns), respectively, and ${\alpha }_{1}$ and ${\alpha }_{2}$ represent the unitless fractional contribution of each component (with ${\alpha }_{1}+{\alpha }_{2}=1$) for a given metabolic coenzyme. It should be noted that ${\tau }_{1}$ and ${\alpha }_{1}$ correspond to free NAD(P)H and ${\tau }_{2}$ and ${\alpha }_{2}$ correspond to enzyme bound NAD(P)H, and that this is reversed in the case of FAD (Fig. 1c)^24^. For each masked pixel within a given image (NAD(P)H or FAD), $m(t)$ was convolved with the corresponding experimentally measured IRF and compared with the temporally binned fluorescence intensity decay data (Fig. 1b). Using the built-in MATLAB function *lsqnonlin*, nonlinear least square analysis was performed with constraints on the lifetime parameters ($0.02 ns \le {\tau }_{i} \le 100 ns$ and $0 \le {\alpha }_{i} \le 1$) and seeding with random initial guesses. The fitting routine was parallelized to handle large FLIM datasets. After processing NAD(P)H and FAD lifetime data through the fitting routine for a given sample, the Fluorescence Lifetime Imaging Redox Ratio (FLIRR) was computed for each masked pixel as:

$$\mathrm{FLIRR}= \frac{{{\alpha }_{2}}_{NAD(P)H}}{{{\alpha }_{1}}_{FAD}}= \frac{\mathrm{fraction}\,\mathrm{of}\,\mathrm{enzyme}\,\mathrm{bound}\,NAD(P)H}{\mathrm{fraction}\,\mathrm{of}\,\mathrm{enzyme}\,\mathrm{bound}\,FAD}$$

where lower FLIRR values have been previously reported to correspond with glycolytic metabolism and higher FLIRR values correspond with a more oxidative phosphorylation (OXPHOS) phenotype (Fig. 1d)^24^.

### Probability density functions of FLIRR populations

Kernel density estimates of the probability density function (PDF) for FLIRR values for each condition were generated by pooling the FLIRR values from each binarized pixel across the three samples and using the MATLAB function *ksdensity* with a normal kernel, unbounded support, and logarithmic boundary correction. For each condition, the probability density function was evaluated from FLIRR values of 0.2–1.3 in increments of 0.01 to represent the measured range of FLIRR values.

### Estimates of differences in FLIRR distributions

The overall differences between the probability density functions were quantified using the overlap index^43^. The overlap index is calculated between two different spheroid treatment conditions by calculating the minimum of two FLIRR probability density functions at each evaluation point (0.2:0.01:1.3) and calculating the sum over that range:

$$Overlap\,Index=\int \mathrm{min}\,\{{pdf}_{x},{pdf}_{y}\}$$

An overlap index value of 1 represents perfect similarity and a value of 0 represents no overlap between the pdfs.

Significance was assessed by computing the median of each FLIRR distribution and computing a two-tailed t-test to compare the differences from Day 0 to Day 3 for each treatment condition at a critical alpha level of 0.05.

### Quantification of proliferation and migration

The total area of each spheroid was quantified and the differences from Day 0 to Day 3 were used as an estimate of proliferation. The total area was calculated by summing every binarized pixel in the segmented spheroid image. The data is displayed as the mean and standard deviation in pixels over the three replicates. Differences across time were assessed for significance by a two-tailed t-test at a significance level of p < 0.05.

**Figure 2:**
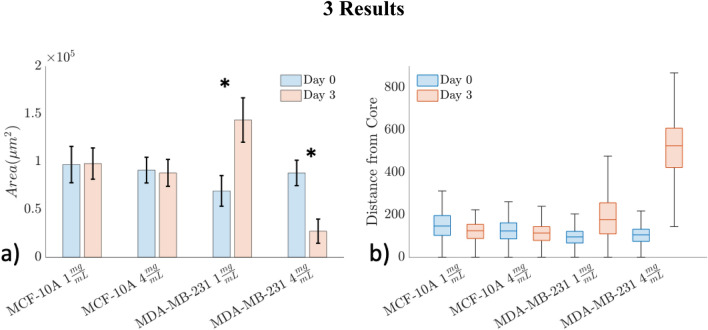
Spheroid morphology over time. (**a**) The total area occupied by spheroids in each group are separated by Day 0 (blue) and Day 3 (red). Each bar represents the mean and standard deviation across the three replicates. For each condition, Day 0 (blue) was compared to Day 3 (orange) using a t-test and significance was indicated with (**a**) * (p < 0.05). The MDA-MB-231 samples had a significant increase in spheroid area at 1 mg/ml and a significant decrease at 4 mg/ml. (**b**) The distance of every pixel from its spheroid center for each condition separated by Day 0 (blue) and Day 3 (red). The whiskers represent 5% and 95%, the box represent 25% and 75%, and median is indicated on the boxplot. The MDA-MB-231 samples had a modest increase in migration distance at 1 mg/ml and a much larger increase at 4 mg/ml.

Migration was estimated by calculating the distance of every binarized pixel with respect to the center of the spheroid (in units of µm). In the case of intact or partially intact spheroids, the center was identified by finding the largest unconnected object and computing the center of mass of that object with the MATLAB function *regionprops*. For samples with only migrating cells (MDA-MB-231 4 mg/ml Day 3), the centers were determined by identifying the centers of voids left behind from previously intact spheroids using overlaid images of NAD(P)H intensity and collagen from SHG imaging. Since there was no collagen where the spheroid was initially embedded, the negative contrast of collagen signal can assist in locating the original spheroid center. After locating the centers, the distance transform was computed relative to the center pixels using the built-in MATLAB function *bwdist* to compute the 2D Euclidean distance transform^44^. The distance distributions were pooled together across all three replicates and plotted in Fig. 2b as boxplots. The whiskers represent 5% and 95%, the box represent 25% and 75%, and horizontal line within the box indicates the median of the distribution.

### 2D spatial analysis of FLIRR

The 2D spatial distribution of FLIRR was adaptively analyzed with consideration of the final organizational status of the spheroids as either completely intact or not. The 2D spatial FLIRR characteristics of completely intact spheroids evaluated FLIRR as a function of Euclidean distance from the edge of the spheroid based on the segmented and binarized spheroid images (Fig. 4). In this analysis, the FLIRR values were evaluated in 15 µm bins from the edge with distances farther than 75 µm approximated as the core. Since the cells at or near the edge of intact spheroids are either in direct contact or proximal to the collagen environment, this 2D FLIRR analysis characterized the impact of collagen proximity on cellular metabolism.

Samples with spheroids that were partially or no longer intact due to the migration of cells lacked defined edges, and thus FLIRR values were analyzed as a function of distance from the centers of what were previously completely intact spheroids. In the case of partially intact spheroids (MDA-MB-231 1 mg/ml Day 3), the center was identified by finding the largest unconnected object with the MATLAB function, *regionprops*, which corresponded to the core, and then computing the center of mass of that object. For samples with spheroids that were no longer intact (MDA-MB-231 4 mg/ml Day 3), the centers were determined by identifying the centers of voids left behind from previously intact spheroids using overlaid images of NAD(P)H intensity and collagen from SHG imaging. Since there was no collagen where the spheroid was initially embedded, the negative contrast of collagen signal can assist in locating the original spheroid center. After locating centers within samples with partially or no longer intact spheroids, the distance transform was computed relative to the center pixels (Fig. 5). This form of 2D FLIRR analysis enabled a spatial assessment of metabolism as a function of migration in bins of 125 µm from the center.

## Results

### Spheroid morphology trends across cell line, collagen concentration, and timepoint

The changes in spheroid morphology were assessed from Day 0 to Day 3 across cell lines and collagen concentrations. MCF-10A spheroids showed no significant differences in area across collagen concentrations or time as shown in Fig. 2a. The MDA-MB-231 spheroids at 1 mg/ml had a significant increase from Day 0 to 3 in area occupied by the spheroid (p = 0.01) suggesting an increase in proliferation, while at 4 mg/ml there was a significant decrease in area occupied by the spheroid (p = 0.0045).

The MCF-10A and the MDA-MB-231 spheroids at Day 0 all had intact cores and approximately the same farthest distance from the core (~ 300 μm), as a reference to compare against non-intact spheroids (Fig. 2b). The MDA-MB-231 spheroids in 1 mg/ml collagen at Day 3 had a moderate increase in spheroid migration relative to Day 0 (farthest distance from the core: ~ 300 μm at Day 0 vs ~ 500 μm at Day 3) while the MDA-MB-231 spheroids in 4 mg/ml collagen at Day 3 had a larger increase in farthest distance from the core relative to Day 0 (~ 300 μm at Day 0 vs ~ 800 μm at Day 3).

### Distinct FLIRR trends across cell line, collagen concentration, and timepoint

**Figure 3:**
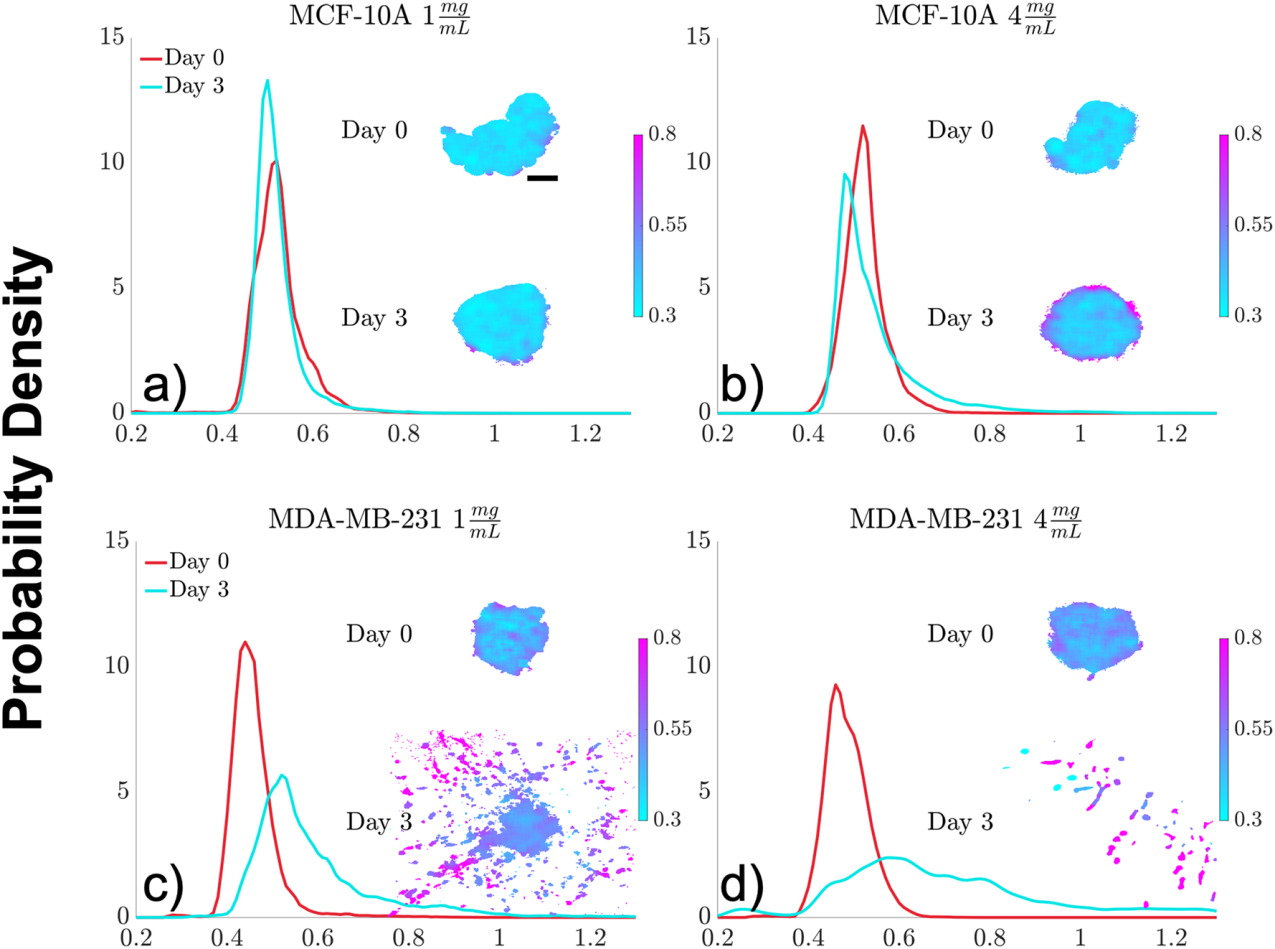
Probability Density Function (PDF) estimates of FLIRR values and representative FLIRR maps. Kernel Density Estimates of PDFs of pooled FLIRR values across the three replicates are shown for MCF-10A at (**a**) 1 mg/ml and (**b**) 4 mg/ml for Day 0 (red) and Day 3 (turquoise) along with a representative example of a FLIRR map with a scale bar representing 100 µm. MDA-MB-231 PDFs are shown at (**c**) 1 mg/ml and (**d**) 4 mg/ml for Day 0 (blue) and Day 3 (orange) along with representative examples of FLIRR maps. The spheroids are all shown over their entire 825 × 825 µm field of view. MCF-10A spheroids demonstrated a small decrease in FLIRR values towards glycolysis with a larger change at 4 mg/ml. MDA-MB-231 spheroids exhibited a large increase in FLIRR values from Day 0 to Day 3, consistent with a shift towards OXPHOS, accompanied with an increase in migration.

The changes in FLIRR values of the spheroids from Day 0 to Day 3, and how those changes varied across cell lines and collagen concentrations were examined using the probability density functions of spheroid FLIRR values (Fig. 3). The MCF-10A spheroids showed a modest shift towards lower FLIRR values from Day 0 to Day 3 in both the 1 mg/ml (overlap index: 0.85, p = 0.54) and 4 mg/ml (overlap index: 0.76, p = 0.85) collagen concentrations (Fig. 3a,b, Fig. S1), which was more pronounced at 4 mg/ml. MCF-10A spheroids in 4 mg/ml collagen on Day 3 had an overall shift towards lower FLIRR values although there was a subpopulation of larger FLIRR values represented by a long tail of the distribution (Fig. 3b), suggesting that a subset of MCF-10A cells were metabolically distinct.

This contrasted with the MDA-MB-231 samples which had a much larger and statistically significant shift towards higher FLIRR values from Day 0 to Day 3. This shift was slightly larger in the 4 mg/ml collagen concentration (overlap index: 0.32, p = 0.05) compared to the 1 mg/ml (overlap index: 0.37, p = 0.02). This may be indicative of an overall shift towards an increased population of invading cells requiring a more energetically efficient energy source such as oxidative phosphorylation. Additionally, MDA-MB-231 spheroids had slightly lower FLIRR values than the MCF-10A spheroids on Day 0, regardless of collagen concentration.

### FLIRR spatial trends as a function of distance from the edge

**Figure 4:**
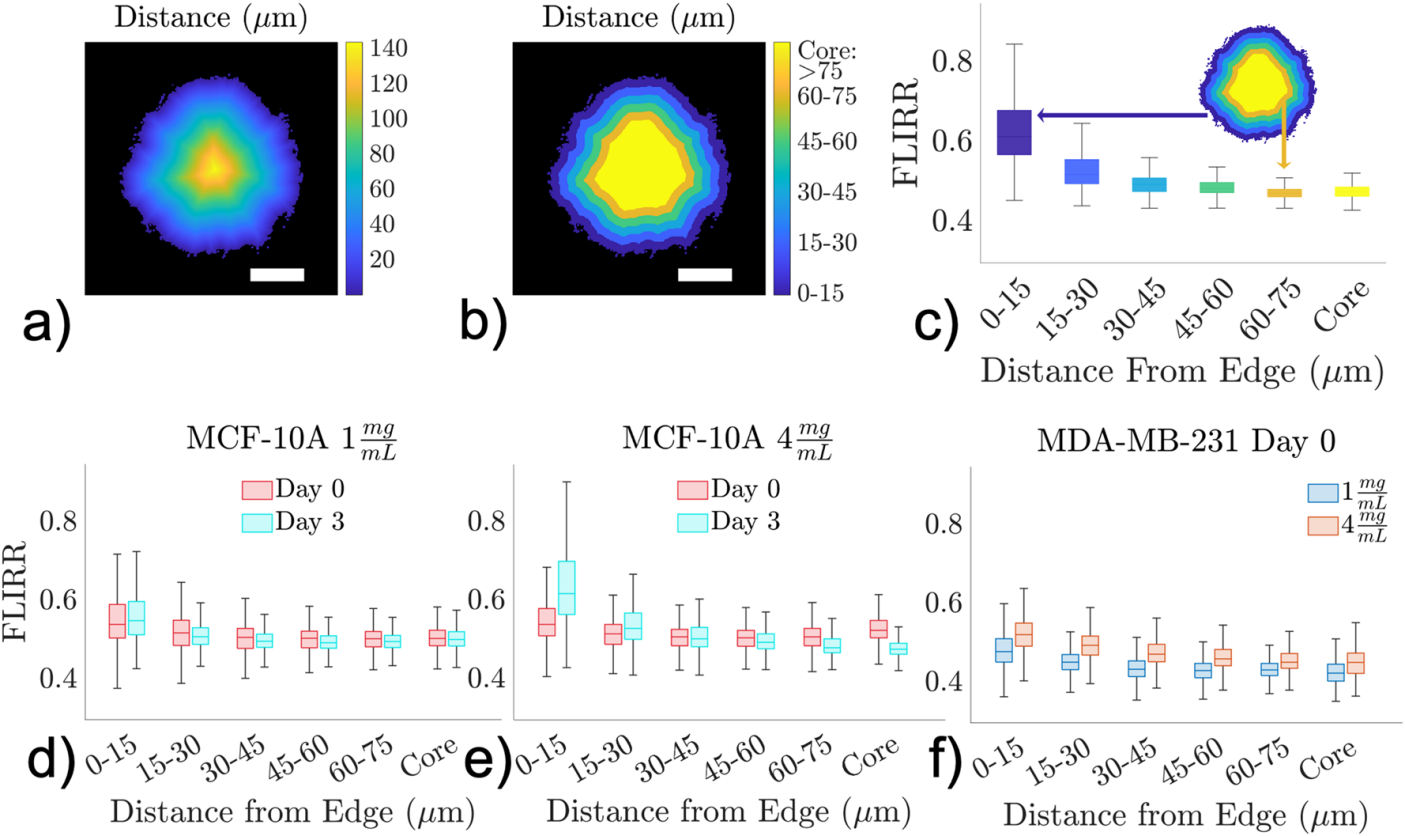
FLIRR values vs distances from the edge. (**a**) Map of Euclidian Distance transform where each pixel’s value represents distance to the nearest edge pixel. (**b**) Discretized distance from the edge map with each color representing a 15um step size. Values greater than 75um are considered as the core. Scale bars (white) indicate 100 µm. (**c**) Representative example of FLIRR vs Distance from the edge plot. For each discretized distance in (**b**) the FLIRR values for those pixels are displayed as a boxplot. Pooled FLIRR vs Distance from the edge for MCF-10A are shown for (**d**) 1 mg/ml and (**e**) 4 mg/ml with Day 0 (red) and Day 3 (turquoise). (**f**) Pooled FLIRR vs Distance from the edge for MDA-MB-231 at Day 0 with 1 mg/ml (blue) and 4 mg/ml (orange). All MCF-10A spheroids and the MDA-MB-231 spheroids at Day 0 displayed enhanced FLIRR values closer to the edge relative to the core with the MCF-10A spheroids on Day 3 at 4 mg/ml displaying the most prominent gradient.

The metabolic spatial gradients across the spheroids were quantified from FLIRR maps to evaluate how they were modified over time, as well as across cell lines and collagen concentrations. All the MCF-10A and MDA-MB-231 spheroids at Day 0 showed pronounced spatial patterns in terms of their FLIRR values as a function of a distance from the spheroid edge in Fig. 4d–f. There were larger FLIRR values in the cells closest to the spheroid edge, while cells closer to the core tended to have smaller FLIRR values. The MCF-10A spheroids in 1 mg/ml collagen displayed little difference between the spatial distribution at Day 0 vs Day 3 (Fig. 4d, Supplemental Fig. S2). The MCF-10A spheroids in 4 mg/ml collagen on Day 3 showed a large increase in FLIRR values at the edge with a decrease in FLIRR values moving towards the core relative to Day 0 (Fig. 4e). These spheroids displayed a large difference in FLIRR distributions between the edge and core (overlap index: 0.18), where the other MCF-10A spheroids had much smaller changes in overlap index (~ 0.5). The subpopulation of the MCF-10A spheroids in the 4 mg/ml at Day 3 compared to Day 0 exhibiting higher FLIRR values (Fig. 3b) was localized at the spheroid periphery where cancer cells are in direct physical contact with the dense collagen matrix. Such heterogenous changes in MCF-10A cells metabolism did not occur in 1 mg/ml collagen (Fig. 3a), thus indicating that the large increase in FLIRR values, indicative of a shift towards OXPHOS, occurs at the invasive front only in 4 mg/ml collagen.

The MDA-MB-231 spheroids at Day 0 in both collagen concentrations displayed relatively similar FLIRR gradients, with elevated FLIRR values near the edge and decreased FLIRR values near the center (Fig. 4f). The MDA-MB-231 spheroids in 4 mg/ml collagen had larger FLIRR values at each distance bin than in the 1 mg/ml samples at Day 0, but both conditions still had approximately the same gradient slope over the distance bins with similar changes in the overlap index near the core (~ 0.4).

### FLIRR spatial trends as a function of distance from centroid

**Figure 5:**
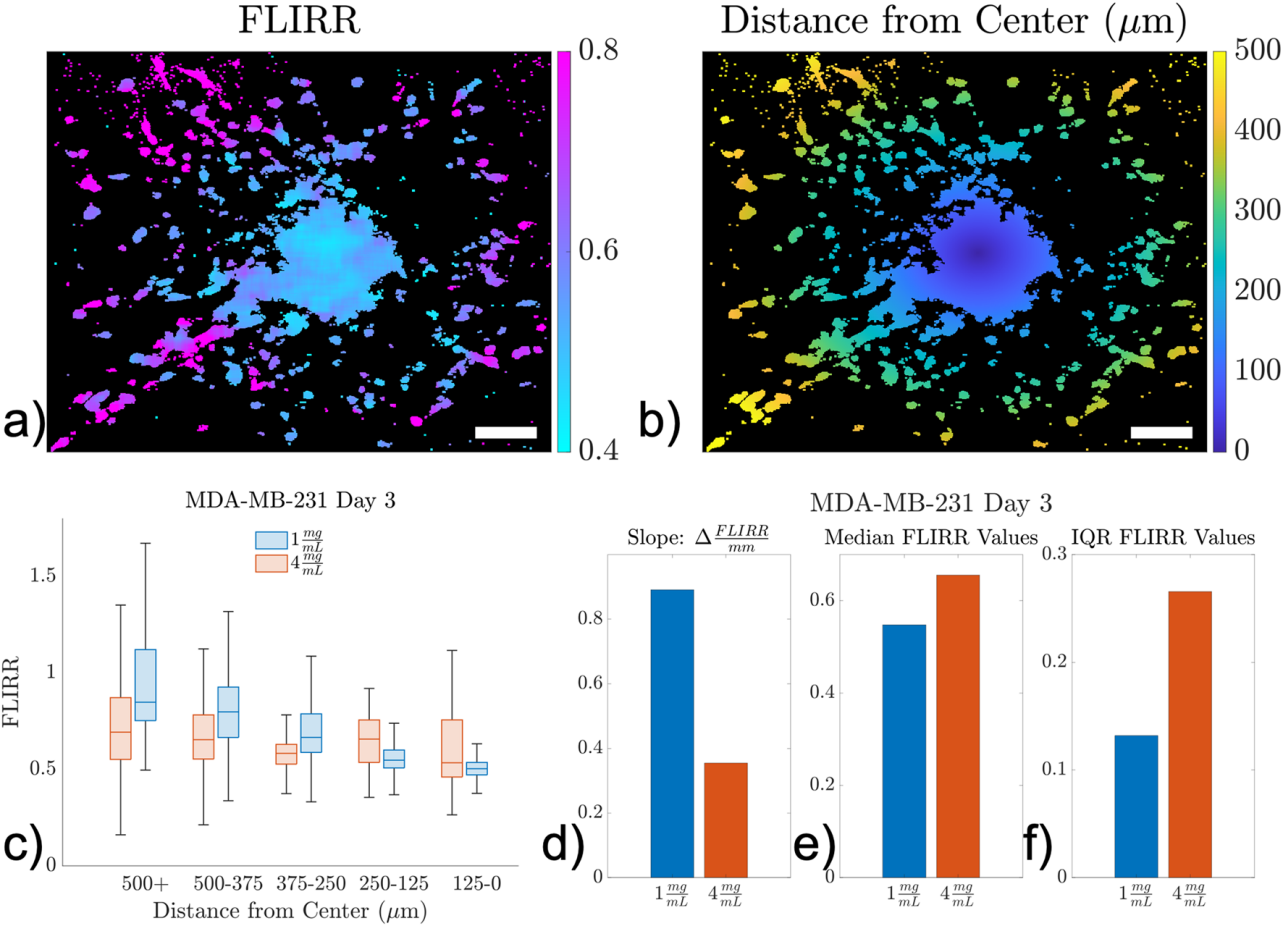
FLIRR gradients as distance from the centroid. (**a**) FLIRR map of an MDA-MB-231 spheroid in 1 mg/ml on Day 3. (**b**) Euclidian distance transform from the centroid of the spheroid where each pixel represents the distance away from the centroid. Scale bars (white) indicate 100 µm. (**c**) FLIRR vs Distance from the centroid plot for MDA-MB-231 at Day 3 comparing 1 mg/ml (blue) vs 4 mg/ml (red). (**d**) Linear slopes fitting the distance from center vs FLIRR values for each collagen concentration: 1 mg/ml (blue) and 4 mg/ml (red). (**e**) Medians of the FLIRR distribution of pooled samples for each collagen concentration. (**f**) Interquartile ranges (IQR) of the FLIRR distributions of pooled samples for each collagen concentration. In these samples, the cells in the 1 mg/ml samples that had migrated the farthest had a preferential shift towards larger FLIRR values consistent with a shift towards OXPHOS in the while the 4 mg/ml samples had a much more spatially heterogenous distribution.

The metabolic spatial gradients in the MDA-MB-231 spheroids at Day 3 were quantified from corresponding FLIRR maps to evaluate how FLIRR values changed as a function of migration distance. MDA-MB-231 spheroids in both collagen concentrations at Day 3 displayed trends of increasing FLIRR values in the cells farthest away from the centroid in Fig. 5c. The 1 mg/ml samples had a much steeper gradient (0.89 FLIRR/mm) than the 4 mg/ml samples (0.35 FLIRR/mm), with the cells farthest away from the centroid having larger FLIRR values and cells closer to the centroid having lower FLIRR values. This was also demonstrated through the overlap index in Supplemental Fig. S3, where the overlap index continues to decrease moving towards the center in both collagen concentrations but in the 1 mg/ml sample the overall change was much more dramatic (overlap index: 1 mg/ml: 0.09 and 4 mg/ml: 0.46).

## Discussion

This work helps to demonstrate the utility of FLIM to non-invasively track the spatial metabolic patterns of breast cancer spheroids in a 3D microenvironment. Importantly, the metabolic profile of each cell line was modulated by collagen concentration and time. The MCF-10A spheroids had a relatively small shift towards smaller FLIRR values over time, while the MDA-MB-231 samples shifted towards larger FLIRR values over time, a shift consistent with OXPHOS, with a more pronounced effect at the higher collagen concentration. Importantly, spatial metabolic gradients were demonstrated across the entire spheroid in both invasive and non-invasive breast cancer cells derived spheroids.

The MCF-10A cell line has been extensively characterized as a model for non-tumorigenic breast tissue^45^. The MCF-10A spheroids shifted towards lower FLIRR values over time, with more prominent shifts occurring at higher collagen concentrations. A shift towards lower FLIRR values has been previously shown to be consistent with a shift towards glycolysis^24^. Other groups have observed breast and lung cancer cells shift towards glycolysis when embedded in higher collagen concentrations^19,46–48^. For example, increases in collagen density induced a metabolic shift in the 3D breast cancer spheroids 4T07 and 4T1 towards increased glucose metabolism and decreased oxygen consumption rate in the tricarboxylic acid (TCA) cycle^48^.

The MDA-MB-231 cell line has been characterized as a highly invasive breast cancer model^30^. The MDA-MB-231 spheroids have lower FLIRR values at Day 0 compared to MCF-10A spheroids, which may be indicative of increased glycolysis, as previously observed through significantly increased lactate production, and decreased mitochondria reduction activity^49^. A low overlap index (~ 0.4) on Day 0 comparing MDA-MB-231 samples with MCF-10A samples, characterized the large baseline differences between the cell lines (Fig. S1).

The MDA-MB-231 samples from Day 0 to 3 displayed significantly different spheroid areas and distance from the core between the 1 mg/ml and 4 mg/ml collagen concentrations (Fig. 2), possibly indicating differences in proliferation and migration in different collagen densities. In the MDA-MB-231 spheroids, the increase in spheroid area in the 1 mg/ml collagen environment from Day 0 to Day 3 (Fig. 2a) potentially indicates that these spheroids were continuing to proliferate at the low collagen concentration. The MDA-MB-231 spheroids on Day 3 demonstrated a farther migratory extent in the 1 mg/ml collagen samples compared to the intact spheroids (~ 500 vs 300 μm), while the 4 mg/ml samples on Day 3 had a substantially larger migratory extent overall (~ 800 μm). These differences in the proliferative and migratory characteristics potentially indicate that the 1 mg/ml collagen MDA-MB-231 samples were a mix of proliferative and migratory phenotypes compared to the 4 mg/ml samples which shifted to a predominantly migratory phenotype.

Proliferating and migrating cancer cells have been shown to have different metabolic demand and rely on different metabolic pathways^9^. In previous work done by Liu et al. the authors described how genetic enrichment analysis revealed that MDA-MB-231 spheroids exposed to stiffer environments with increased migratory phenotypes had an upregulation of OXPHOS and fatty acid metabolism genes and downregulation of glycolysis genes compared to softer environments^28^. This is consistent with the increase in FLIRR observed for MDA-MB-231 spheroids at Day 3 with a larger increase in FLIRR in the 4 mg/ml samples with the greater cell migration compared to the lower collagen concentration.

Epithelial cells, like MCF-10A, experience contact inhibition which normally inhibits proliferation when the cell density reaches a threshold^50^. However, a stiff ECM has shown to be able to downregulate this contact inhibition and induce a malignant phenotype in MCF-10A cells through YAP/TAZ activation^50,51^. This malignant transformation may also explain the overall shift towards lower FLIRR values (Fig. 3a,b) by starting to induce the Warburg effect and shift towards glycolysis^7,8^.

Interestingly, while there was an overall small shift towards lower FLIRR values in the MCF-10A samples from Day 0 to Day 3 (Fig. 3a,b), the cells closest to the edge had an increase in FLIRR values (Fig. 4d,e). In the 4 mg/ml MCF-10A sample at Day 3, this trend was particularly enhanced. The cells closest to the edge of the spheroid would be directly engaging with the collagen and may represent cells in a pre-invasive phenotype^51^. The invasion transformation of MCF-10A cells may take longer than the timepoint of Day 3 when the spheroids were imaged^31^. Similarly, MDA-MB-231 cells at Day 0 in both collagen concentrations had lower values closer to the core and elevated FLIRR values towards the edge (Fig. 4f), which may be starting to shift from a purely proliferative phenotype to a migratory phenotype^52,53^.

In the MDA-MB-231 spheroids on Day 3, there were increasing FLIRR values as a function of distance away from the core (Fig. 5c). In the 1 mg/ml samples, the cells that had travelled the farthest from the core had a larger increase in FLIRR values, while cells closer to the core had lower FLIRR values. In comparison, the 4 mg/ml MDA-MB-231 spheroids at Day 3 had a more modest FLIRR gradient as a function of distance from the core (Fig. 5d). For the 1 mg/ml samples the gradient represented the change in cells from the remaining core to cells invading into the collagen. However, for the 4 mg/ml spheroids there was no longer an aggregation of cancer cells at the core. These 4 mg/ml samples consequently were much more heterogenous with overall larger median FLIRR values combined with a larger IQR, resulting in a flatter distribution both overall and spatially.

Prior work has found that the leading invasive edge of cancer spheroids shift their metabolism towards OXPHOS to meet the energy demands of invasion^9,12,53^. Other groups have shown that MDA-MB-231 migration in stiffer collagen gels corresponds to an increase in glucose uptake and ATP/ADP ratio^11^. Cancer cells on the leading edge of migration have been shown to have increased number of mitochondria and ATP output^52^. The leader cells of those migrating cells had increased metabolic output compared to the follower cells and may explain the FLIRR gradients as a function of distance from the center. Additionally, Davis et al. showed that primary MDA-MB-231 tumor cells were significantly more glycolytic and underwent less OXPHOS compared to the matched micrometastatic cells using single-cell RNA sequencing, flow cytometry and qPCR^54^. In that work, inhibiting OXPHOS in MDA-MB-231 cells inoculated in mice significantly decreased the number of metastases found in the lungs demonstrating the need for OXPHOS in the migration cascade. Understanding the phenotypic metabolic changes underlying cancer cells’ migration transformation would be critical to develop metabolic therapies that are able to target aspects of the metastatic cascade^55^.

This work did not confirm the differences in metabolic profiles and proliferation through ex vivo molecular analysis techniques such as RNA sequencing. The exact stiffness of the 1 mg/ml and 4 mg/ml collagen concentrations was not measured; however, prior work has shown collagen concentration correlates with rheology measurements of stiffness^19^. This study did not examine the differences in collagen structure and orientation due to limited spatial resolution. Additionally, our model of ECM consisted of only collagen and did not include other critical features of the ECM such as fibroblasts. There were only a limited number of replicate spheroids per condition (n = 3). Statistical tests were not done to compare the differences between pooled sample distributions across conditions because pixels were spatially binned and are subsequently correlated, breaking the assumption of independence.

In summary, this work demonstrated that FLIM can non-invasively monitor the spatial metabolic profile of live 3D breast cancer spheroids in relevant and tunable microenvironments. The MCF-10A and MDA-MB-231 samples differentially shifted their metabolic profile in response to modifications to the collagen concentration of the ECM. The physical properties of the ECM (e.g., density) also impacted the proliferative versus migratory phenotype of MDA-MB-231 cells. Finally, FLIM was able to reveal spatial metabolic gradients across individual spheroids. FLIM’s ability to non-invasively probe the metabolic profile while preserving the spatial information makes it a critical tool to longitudinally track metabolic changes in 3D samples.

## Supplementary Information

Supplementary Figures.

## Data Availability

The datasets generated during and/or analyzed during the current study are available from the corresponding author on reasonable request.
