# Supplementary material for: Fluorescence Lifetime Imaging Microscopy (FLIM) reveals spatial-metabolic changes in 3D breast cancer spheroids
Source: Sci Rep. 2023 Mar 3;13:3624. doi: 10.1038/s41598-023-30403-7 (PMC9984376; doi:10.1038/s41598-023-30403-7)
Supplement: Supplementary file 1 — Supplementary Figures. [file 41598_2023_30403_MOESM1_ESM.docx]

## Supplementary Information

# **Fluorescence Lifetime Imaging Microscopy (FLIM) reveals spatial-metabolic changes in 3D breast cancer spheroids**

Kavon Karrobi^a,1^, Anup Tank^a,1^, Mohammad Ahsan Fuzail^a^, Madhumathi Kalidoss^a^, Karissa Tilbury^b^, Muhammad Zaman^a^, Jacopo Ferruzzi^a, c^, Darren Roblyer^a *^


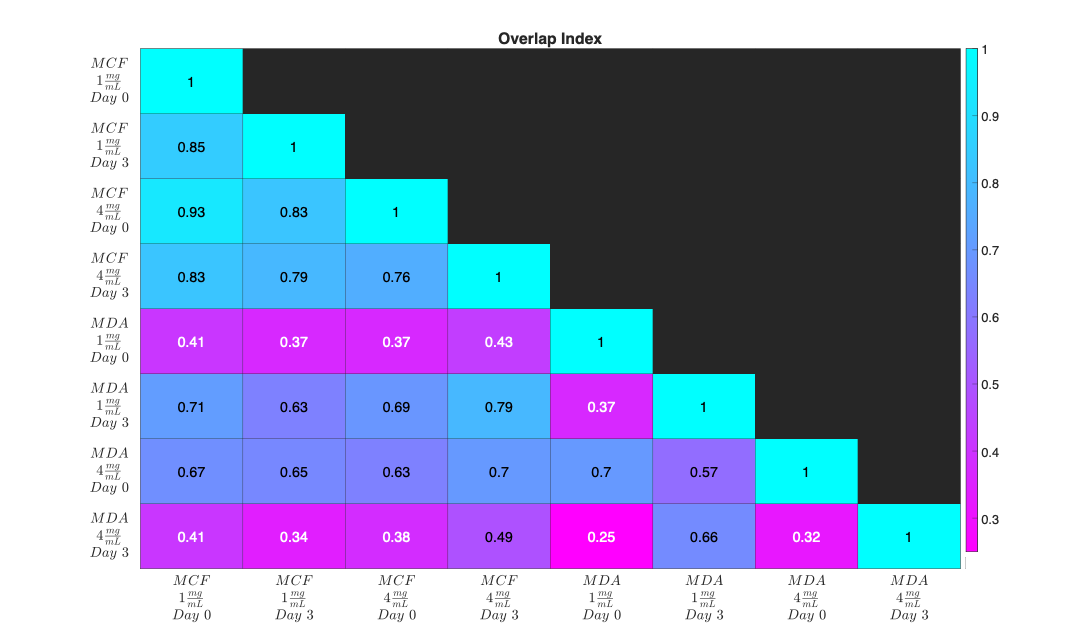


Figure S1 Overlap Index Heatmap. The overlap index is computed for the FLIRR distributions of each unique combination of cell line, collagen concentration and timepoint and displayed with its value and the color coded.


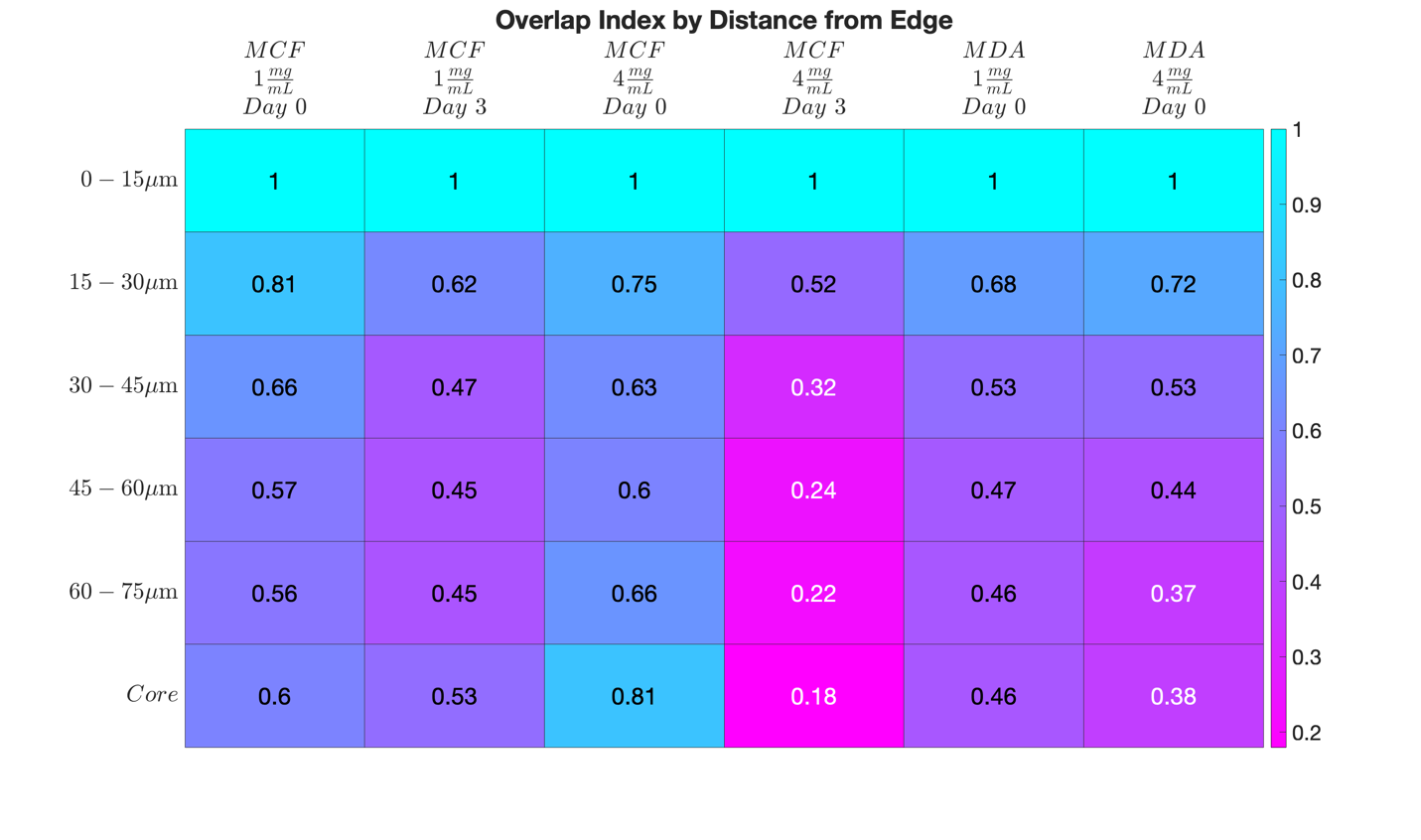


Figure S2 Overlap Index by Distance from Edge Heatmap. The overlap index by distance from the edge is computed for the FLIRR distributions of intact spheroids for each unique combination of cell line, collagen concentration and timepoint relative to the 0-15 um bin.


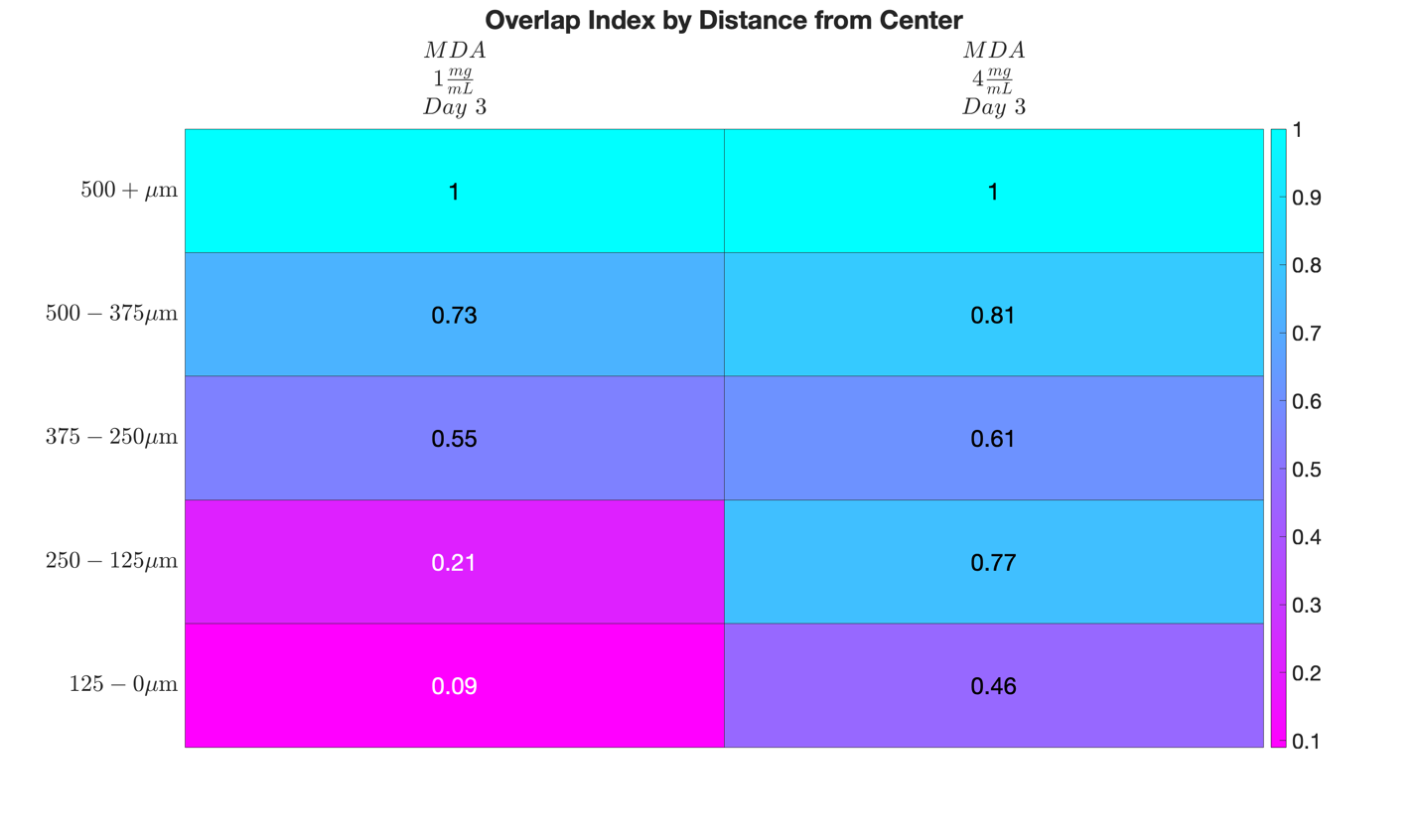


Figure S3 Overlap Index by Distance from Center Heatmap. The overlap index by distance from the center is computed for the FLIRR distributions of migrating spheroids for each unique combination of cell line, collagen concentration and timepoint relative to the 500+ um bin.
